# 

**Published:** 1888-10

**Authors:** 


### BOOKS AND PAMPHLETS RECEIVED.

Beitrag zur Entwickelungs- und Lebensweise der Aphiden, von Dr. H. F. Kessler. No. 3, Bd. XLVII. der Ksl. Leop.-Carol. Deutschen Akademie der Naturforscher.

Memoria de la Secretaria de Gobernacion Policia y Formento. San Jose, Costa Rica, 1888

Contributions to the Natural History of Alaska. By L. M. Turner. No. II. Article series of publications issued in connection with the Signal Service U. S. Army. 4to. Washington, 1886.

Annual Report of the Board of Regents of the Smithsonian Institution. Part II., 1885. 8vo. Washington, 1886.

Report of the Commissioner of Agriculture. 1887. 8vo. Washington, 1888.

Preliminary Abstract Report of the Marine Laboratory, University of Pennsylvania.

Swine Plague; its Causes, Nature and Prevention. By Frank S. Billings, Director of the Patho-Biological Laboratory University of Nebraska. 8vo. Lincoln.

Documents Issued by the State Board of Health through Henry B. Baker, M.D., Secretary. Lansing, Mich.

Announcement of Gross Medical College of Denver. Session 1888–89.

ANNUAL ANNOUNCEMENT OF THE NEW YORK COLLEGE OF VETERINARY SURGEON AND SCHOOL OF COMPARATIVE MEDICINE. Session 1888–89.

CHICAGO VETERINARY COLLEGE. Prospectus for Session 1888–89.

CONSTITUTION AND BY-LAWS OF THE NEW JERSEY STATE VETERINARY SOCIETY. Paterson, 1888.

THE HORSE'S FOOT AND HOW TO SHOE IT. By John Airth, M. R. C. V. S. Goldie Bros. Sioux City, Iowa.

THE STERNUM IN THE SOLITARY SAND-PIPER, AND OTHER NOTES. By R. W. Shufeldt, M.D. Reprint from the Auk, Vol. X., No. 3.

DESCRIPCION DE UNA ESPECIE NEUVA DE "GALLINA DE MONTE." Por Jose C. Zeledon.

### AMERICA (NORTH).

### CANADA.

CAP ROUGE.—Le Naturaliste Canadien.
OTTAWA.—The Ottawa Naturalist.
TORONTO.—Medical Science.

### MEXICO.

MEXICO.—La Naturaleza. Tomo I., No. 1–3.

### UNITED STATES.

ALBANY.—Medical Annals.
AUGUSTA.—The Sanitary Inspector.
BALTIMORE.—Johns Hopkins' University Circulars.
CHAMPAIGNE.—Bulletin Illinois State Laboratory of Natural History. Vol. II., Art. 8, Vol III., Art. 4.
CHICAGO.—Weekly Drovers' Journal, National Live Stock Journal, Chicago Tribune, The Prairie Farmer.
DENVER.—Proceedings Colorado Scientific Society Vol. II., Part 3.
DETROIT.—The Microscope.
KNOXVILLE.—Agricultural Science.
NEW YORK.—American Veterinary Review, New York Medical Journal, Medical Analectic, Scientific American, Forest and Stream, Annual Report of the Trustees of the American Museum of Natural History. 1887–88.
PHILADELPHIA.—Medical and Surgical Reporter, Therapeutic Gazette, American Naturalist, Reins and Whip.
RED WING.—Public Health in Minnesota.
ST. LOUIS.—Weekly Medical Review.
WILMINGTON.—Delaware Farm and Home.

### AMERICA (CENTRAL).

### COSTA RICA.

SAN JOSE.—Annales des Museo Nacional. Tome I.

### ASIA.

### CHINA.

SHANGHAI.—Journal of the China Branch of the Royal Asiatic Society. No. 5, 1887.

### INDIA.

BOMBAY.—Journal of Natural History Society. Vol. III., No. 2–3.

MADRAS.—The Quarterly Journal of Veterinary Science in India. July, 1888.

### JAVA.

BATAVIA.—Natuurkundig Tijdschrift voor Nederlandsch-Indie. Deel XLVII., Veeartsenijkundige Bladen voor Nederlandsch-Indie. Deel III., Nos. 1–2.

### AUSTRALIA.

### NEW SOUTH WALES.

SYDNEY.—Proceedings of the Linnean Society, Vol. II., Part 4; Vol. III., Part 1. Journal and Proceedings of the Royal Society. Vol. XXI.

### VICTORIA.

MELBOURNE.—Annual Report of the Zoological and Acclimatization Society. 1887.

### EUROPE.

### AUSTRIA.

VIENNA.—Annalen des K. K. Natur-historischen Hofmuseums. Bd. III., No. 2. Verhandlungen der K. K. Zoologisch-botanischen Gesellschaft. Band XXXVIII., Quar. 1–2. Ornis, July, 1888. Mittheilungen des Ornithologischen Vereins. Jahr. XII., Nos 6–8. Oesterreichische Monatschrift fur Thierheilkunde.

### BELGIUM.

BRUSSELS.—Annals de Medicine Veterinaire.

GHENT.—Annales et Bulletin de la Societe de Medicine.

### DENMARK.

COPENHAGEN.—Tidsskrift fur Veterinærer.

### FRANCE.

AMIENS.—Bulletin Mensuel Societé Linneene du Nord de la France. Tome IX., Nos. 187–190.

LYONS.—Journal de Medicine Veterinaire et de Zootechnie.

MARSEILLES.—Annales du Musee d'Historie Naturelle, Zoologie. Tome I.–II. 1882–85.

PARIS.—Le Progres Medical, Recueil de Medicine Veterinaire, Bulletin de la Societe Zoologique. Vol. XIII., Parts 1–7.
TOULOUSE.—Revue Veterinaire.

GERMANY.

AUGSBURG.—Wochenschrift fur Thierheilkunde und Viehzucht.
BERLIN.—Archiv fur Wissenschaftliche und Praktische Thierheilkunde. Sitzungs-Berichte der Gesellschaft Naturforschender Freunde. 1880–87.
BERN.—Verhandlungen des Natur-historischen Vereines. 1888.
BRAUNSCHWEIG.—Jahresbericht des Vereins fur Natur-wissenschaft.
DRESDEN.—Jahresbericht der Gesellschaft fur Natur-und Heilkunde. 1888.
FRANKFURT.—Der Zoologische Garten.
HALLE.—Leopoldina, Heft 23; Thiermedizinische Vortrage, Bd. I., Heft 3.
JENA.—Zeitschrift von der Medizinisch Natur–wissenschaftlichen Gesellschaft. Bd. XXII., Heft 1–2.
KARLSRUHE.—Thierarztliche Mittheilungen. Amtliche Bekannlmachungen uber das Veterinariwesen im Grossherzogthum. 17 Jahr., No. 7–13.
STUTTGART.—Repertorium der Tierheilkunde, Hippologische Revue.

GREAT BRITAIN.

LONDON.—The Veterinary Journal, The Veterinarian.

ITALY.

MILAN.—La Clinica Veterinaria.
PALERMO.—Il Naturalista Siciliano. Anno VII., Nos. 10–11.
PAVIA.—Bollettino Scientifico. Anno X., Nos. 1–2.
PISA.—Giornale di Anat., Fisiol. e Patol. Degli Animali.
TURIN.—Bollettino dei Museé di Zoologia ed Anatomia Comparata. Vol. III., Nos. 44–48; Il Medico Veterinario.
UDINE.—Giornale di Veterinaria Militare.

NETHERLANDS.

HAGUE.—Verslag van der Tœstand van het Kon. Zool. Bot. Genootschap. 1887.

RUSSIA.

ST. PETERSBURG.—Archives of Veterinary Science

SPAIN.

MADRID.—Gaceta Medico-Veterinaria.
VALENCIA.—La Cronica Medica.

SWITZERLAND.

ZURICH.—Schweizer-Archiv fur Thierheilkunde.

